# Octacalcium Phosphate Solubility Product from 4 to 37 °C

**DOI:** 10.6028/jres.093.153

**Published:** 1988-10-01

**Authors:** M. S. Tung, N. Eidelman, B. Sieck, W. E. Brown

**Affiliations:** American Dental Association Health Foundation, National Bureau of Standards Gaithersburg, MD 20899

**Keywords:** equilibrium constants, hydrolysis, octacalcium phosphate, solubility, solubility product

## Abstract

Octacalcium phosphate (OCP) is proving to be an important intermediary in the formation of tooth and bone mineral and various pathological calcifications. Before this mineral can form, its solubility product must be exceeded. Thus, a knowledge of its precise values under various conditions is required for a basic understanding of calcification processes.

The methodology suitable for measuring the solubility of metastable phases was developed and used to determine the negative logarithms of the solubility products of OCP, p*K*_sp_(OCP), at 4, 4.8, 6, 18, 23.5, and 37 °C. This methodology includes (1) the use of high solid-to-liquid ratio, 10 mg/mL, to minimize the effects of hydrolysis, (2) frequent sampling during equilibration to detect possible effects of hydrolysis, (3) equilibration from supersaturation and from undersaturation, and (4) equilibration in the absence and presence of a CO_2_-containing atmosphere.

The resulting p*K*_sp_(OCP) values are 48.3±0.2, 48.3±0.2, 48.2, 48.3, 48.4±0.1 and 48.7±0.2 at 4, 4.8, 6, 18, 23.5, and 37 °C. A 5.5% CO_2_ atmosphere did not change the apparent p*K*_sp_(OCP) value significantly. The value of p*K*_sp_(OCP) obtained by approaching equilibrium from supersaturation was essentially the same as that from undersaturation.

The effects of (1) the use of different ionic models, (2) OCP hydrolysis, and (3) differences in equilibrium constants on the apparent p*K*_sp_(OCP) values are described; the latter two contribute significantly to the differences in p*K*_sp_(OCP).

## Introduction

Octacalcium phosphate (OCP), Ca_8_H_2_(PO_4_)_6_·5H_2_O, is frequently encountered in calcium phosphate systems more basic than dicalcium phosphate dihydrate (DCPD), CaHPO_4_·2H_2_O. It forms and hydrolyzes rapidly under physiological conditions [1]. There are many indications that it is the precursor in the formation of tooth and bone minerals and pathological calcium phosphates [2,3]. The structure of OCP has been determined [4] and its solubility reported [5–8]. The recently published values [5,6] for p*K*_sp_(OCP) (p*K*_sp_(OCP)= −log *K*_sp_(OCP), where *K*_sp_(OCP) is the solubility product of OCP) are in variance with older values [7,8] as shown in table 1. Thus, it is important to know the precise thermodynamic solubility of OCP in the form of the solubility product [p*K*_sp_(OCP)] in order to assess the degree of saturation of biological fluids with respect to OCP. We report here values for p*K*_sp_(OCP) at 4, 4.8, 6, 18, 23.5 and 37 °C. One of the main difficulties in obtaining the solubility of OCP is that OCP is not the most stable phase and will hydrolyze to other thermodynamically more stable calcium phosphates. Therefore, procedures suitable for measuring the solubility of metastable phases were developed and used. The methods minimize the effect of OCP hydrolysis and include (1) the use of high solid-to-liquid ratio, (2) frequent sampling during equilibration to detect possible effects of hydrolysis, (3) equilibration from supersaturation and from undersaturation, and (4) equilibration in the absence and presence of a CO_2_ containing atmosphere, since carbonate slows down the hydrolysis. We have also studied the effect of OCP hydrolysis on the apparent solubility product and examined the possible causes for the differences in OCP solubility constants in the literature as compared to the values reported here.

## Materials and Methods

### Materials

OCP was prepared by two methods: (I) Hydrolysis of DCPD slurry in distilled water with addition of water dropwise over a period of about 3 months at room temperature [9]; this sample was designated as OCP-A and used for most of the study unless specified otherwise. The DCPD that was used for preparing the OCP was prepared by ammoniating an aqueous solution initially saturated with monocalcium phosphate monohydrate (MCPM), Ca(H_2_PO_4_)_2_·H_2_O, and DCPD, (i.e., at the singular point of MCPM and DCPD) according to the procedure described by Moreno et al. [10]. The OCP had a Ca/P ratio of 1.33±.02 and yielded a characteristic OCP x-ray diffraction pattern. (2) Growth of OCP by the constant composition method at pH=6[11]. This preparation was designated as OCP-B.

### Analytical Methods

Two spectrophotometric methods were used to determine the concentration of calcium and phosphate: calcium as the Ca-Arzenazo III complex [12] and by atomic absorption [13], phosphate as vanado-molybdate [14] complex or the phosphomolybdate malachite green complex [12]. The estimated errors were ± 1.5% of the amounts analyzed. The pH was measured with a combination glass-calomel reference electrode with estimated errors of ±0.008 pH units.

### Equilibration Procedures

The saturated solutions of OCP were obtained by allowing OCP crystal dissolution or growth to proceed to equilibrium in solutions initially under-saturated or supersaturated with respect to OCP. Crystals of OCP were added to various concentrations of phosphoric acid (10 mg/mL), when equilibrium was approached from undersaturation, or to solutions containing potassium dihydrogen phosphate and calcium nitrate, when equilibrium was approached from supersaturation. The suspensions were stirred with a magnetic stirrer, and the experiments were carried out either in covered jacketed Pyrex cells through which thermostated water was circulated or in sealed plastic tubes which were mounted in a constant temperature bath at 4,4.8,6, 18, 23.5, or 37 °C. In some of the experiments the nitrogen or 5.5% CO_2_/94.5% N_2_ mixture presaturated with water was bubbled through the solutions. The equilibrations were allowed to proceed for different periods of time (15 min to 3.5 months). Samples were withdrawn periodically from the suspensions and either centrifuged at 15,000 rpm in an Eppendorf centrifuge (No. 5414) or filtered through a 0.22 *μ*m filter (Millipore, Bedford, MA).^1^

The pH was measured just before taking the samples, after separation of the solids, and, in some cases, continuously at the initial stage. The calcium and phosphate concentrations of the supernatants or filtrates were determined as described above.

The ion activity products for OCP [*IP*(OCP)] were obtained from the calcium and phosphate concentrations, pH and ionic strengths using a computer program [15] which calculates the ion activity coefficients through the use of the extended Debye-Hückel equation [19] or Davies equation [20] and takes into account the ion pairs CaOH^+^, CaHPO_4_, ${\text{CaH}}_{2}{\text{PO}}_{4}^{+}$, CaCO_3_ and ${\text{CaHCO}}_{3}^{+}$ [16], and dissociation of H_3_PO_4_ and H_2_O, (Appendix) as follows:

$$IP(\text{OCP})={\left({\text{Ca}}^{2+}\right)}^{4}(H^{+}){\left({{\text{PO}}_{4}}^{3-}\right)}^{3}.$$

Here parentheses indicate ionic activities. The *K*_sp_(OCP)=*IP*(OCP) when the solution is in equilibrium with OCP. Unless specified otherwise, the extended Debye-Hückel equation was used.

## Results

The compositions of solutions equilibrated with two different preparations of OCP and p*IP*(OCP) values as a function of time at 37 °C in a nitrogen atmosphere are presented in tables 2 and 3 (table 2 for solutions initially undersaturated, and table 3 for solutions initially supersaturated with respect to OCP). The results are similar for both preparations whether or not the initial solutions are under or super saturated with respect to OCP, except after 10 days. Equilibrium was obtained rapidly from both undersaturation and supersaturation (within 15 to 20 min) as indicated by constancy of the values of p*IP*(OCP) and pH of the solutions. In the equilibration of OCP-A from undersaturation, the p*IP*(OCP) was constant for about 10 days (depending on the initial phosphoric acid concentration) and then increased slowly after that. This increase is attributable to the hydrolysis of OCP to a more basic product which has lower solubility than that of OCP under the conditions studied. The pH of the solution increased rapidly in the first 15 minutes due to the dissolution of OCP, remained constant for 4.5 h, and then decreased slowly over the range of time studied, as indicated in table 2. The latter decrease can only be due to the hydrolysis of OCP to a non-stoichiometric “apatite.” The hydrolysis produces phosphoric acid approximately as follows:

$$(10-x){\text{Ca}}_{8}{\left({\text{PO}}_{4}\right)}_{6}H_{2}\cdot 5H_{2}O\to 8{\text{Ca}}_{10-x}{\left({\text{PO}}_{4}\right)}_{6}{\left(\text{OH}\right)}_{2-2x}+(12-6x)H_{3}{\text{PO}}_{4}+(34+11x)H_{2}O.$$

The Ca/(P−P_0_) ratios in solutions should indicate the dissolution ratios. These ratios, aithough varying (rom the ratio for congruent dissolution (4/3), decreased with time, indicative of a hydrolysis process which increased the phosphate concentration in the solution. After equilibration of sample OCP-A for 3.5 months, only apatite lines could be detected in the x-ray powder diffraction patterns, and the p*IP*(OCP) was 50.1. When equilibrium was approached from supersaturation, the pH of the solution decreased rapidly from 5.7 to 5.47 in the first 20 minutes, as indicated in table 3, due to the precipitation of calcium and phosphate from solution and crystal growth of OCP. After that, the pH of the solution decreased slowly, and yet the p*IP*(OCP) remained constant. The slow decrease in the pH of the solution is again indicative of OCP hydrolysis to an apatite-like phase. Al-though the hydrolysis of OCP-A seemed to occur early, as indicated by the decrease in pH and ΔCa/ΔP in 23 h, the effects of hydrolysis on the value of p*IP*(OCP) was observed only after 10 days in the equilibration from undersaturation (table 2). The hydrolysis of OCP-B was slow compared to that of OCP-A, as indicated by the change in pH of the solution (table 2); therefore, the variation of p*IP*(OCP) with time was not as great as in the case of OCP-A.

The results of p*K*_sp_(OCP) under a 5.5% CO_2_ atmosphere or under air (tables 4 and 5) fall in the same ranges as those under N, (tables 2 and 3). The hydrolysis rate, as indicated by decreases in pH and ΔCa/ΔP in tables 2, 4, and 5, depends on the CO_2_ content in the atmosphere and decreases in the order N_2_>air>5.5% CO_2_ due to the inhibitory effect of carbonate on hydrolysis. On the other hand there is no observable effect of carbonate on the p*K*_sp_(OCP) presumably because carbonate cannot be incorporated into the OCP crystals.

The solubility products under different initial solution compositions at 4, 6, 4.8, 23.5, and 37 °C are shown in tables 6–11 together with the equilibrium times, composition of solutions and ΔCa/ΔP. The results indicate that hydrolysis is slower when the initial solution has lower pH (i.e., higher phosphoric acid concentration), lower temperature, or the initial solution is supersaturated. There are indications of lack of attainment of saturation in the early stages of the equilibration in the presence of CO_2_ (table 4) and in the solutions with low initial pH (table 11); the relatively high values of p*IP*(OCP) in the longest equilibrations indicate that the rate of hydrolysis approximated that of dissolution. The p*IP*(OCP) values obtained from the solutions which had not reached saturation in the early stages of the equilibration and from the solutions in which hydrolyzed OCP had increased the value of p*IP*(OCP) were not included in the results shown in table 1. The solutions were not considered to be in equilibrium with OCP when the p*IP*(OCP) values of the initial solutions or the final solutions were 0.2 units higher than the average p*K*_sp_(OCP) value.

Our values of p*K*_sp_(OCP) (second column of table 1) increased only slightly in the temperature range 4 to 37 °C. Those reported by Shyu et al. [5] and Heughebaert and Nancollas [6] also varied only slightly in the range from 25 to 45 °C. Considering the experimental errors and the uncertainty of equilibrium constants used, these two studies indicate that the solubility product of OCP is not affected significantly by temperature. The two sets of data in the temperature range where they overlap are similar after correction for differences in ionic models and equilibrium constants, although our values for p*K*_sp_(OCP) are smaller than theirs. An attempt is made in the discussion to compare the effect of OCP hydrolysis and the different ionic models and equilibrium constants on the p*K*_sp_(OCP) values.

## Discussion

The OCP is not the most stable phase under the conditions studied here or reported in the literature. Thermodynamically it will hydrolyze to other calcium phosphates; the kinetic rates depend on temperature, pH, solid-to-liquid ratio, and calcium-to-phosphate ratio in solution. Similar problems of instability have also been encountered in the solubility studies of carbonate- and fluoride-containing apatites [17] and tetracalcium phosphate. The longitudinal data given in tables 2–5 and 11 clearly reveal that hydrolysis was taking place in these studies: (l) The pH values of the solutions decreased monotonically with time. This is the result of OCP hydrolyzing into a more basic salt. In several instances, the pH changed more than a unit in its value without a large change in the p*IP*(OCP). The lack of change in the value of p*K*_sp_(OCP) is apparently related to the hydrolysis process being slow compared to the rate of dissolution of OCP when relatively high solid-to-solution ratios are used. (2) The calcium and phosphate concentrations increased with time after equilibrium had been reached. This is in accord with the phase diagram for OCP. Despite the relatively large changes in pH and calcium and phosphate concentrations, the p*IP*(OCP) remained quite constant in most in-stances. This is in accord with a process in which the composition shifts along the OCP isotherm toward higher concentrations and lower pH values [18] so that the solution remains essentially saturated. One reason for this behavior is our use of a relatively high solid-to-solution ratio in the equilibrations; this favors a high dissolution rate compared to the hydrolysis rate.

Heughebaert and Nancollas [6] state: “The mean molar calcium/phosphate ratios of the solid phases after equilibrium in these experiments were 1.30±0.05 at 25°C and 1.33 ±0.02 at 45°C confirming the good stability of OCP in the aqueous media under these conditions of temperature and pH. In particular, hydrolysis of OCP into an HAp-like phase at 45°C in the pH range 5.5–6.8 was never detected under the experimental conditions used.” The changes in the solution composition at 25°C [6], which is much more sensitive to the hydrolysis as compared to the changes in Ca/P ratios of the solid, indicate some hydrolysis of OCP. The ΔCa/ΔP values calculated from the Heughebaert and Nancollas 25°C data (table II, ref. [6]) reveal that the ΔCa/ΔP ratios were larger than 1.33 (table 12) when equilibrium was approached from supersaturation (av ΔCa/ΔP= 1.53 when the unreliable value from expt. 52G is omitted) and all the ΔCa/ΔP ratios were smaller than 1.33 when equilibrium was approached from undersaturation. There is no overlapping in the two sets of data. The above results are in accord with hydrolysis processes in which a product more basic than OCP is being formed. For example, consider a process in which precipitation of OCP alone is occurring. The calcium lost from solution divided by phosphate that is lost in the same time interval, ΔCa/ΔP, would be 1.33. Now if, say, OHAp precipitated along with the OCP (or was formed by hydrolysis of OCP in situ), the quantity ΔCa/ΔP would be larger than 1.33. This is the situation with most of the 25°C data from supersaturation (table 12). The reverse would be the case when approaching equilibrium from undersaturation. If only OCP were dissolving, ΔCa/ΔP would be 1.33. But if some OHAp were forming, Ca and P would be removed in a ratio greater than 1.33, leaving a net dissolved ΔCa/ΔP less than 1.33. Thus, the ΔCa/ΔP data at 25°C (table 12) indicate that hydrolysis of OCP had occurred. The ΔCa/ΔP ratios calculated from the data at 37°C by Shyu et al. [5] also indicate the occurrence of OCP hydrolysis. On the other hand, the solution compositions of the 45°C equilibrations [6] do not indicate that hydrolysis of OCP had occurred, and the p*K*_sp_(OCP), calculated from these data using our ionic model, is 48.6±0.4, the same as our value at 37°C.

The p*K*_sp_(OCP) values for four groups of investigators using different ionic models and equilibrium constants are compared in table 1. It is apparent that the p*K*_sp_(OCP) values reported by Heughebaert and Nancollas [6] and by Shyu et al. [5] at 25 and 37°C are about an order of magnitude higher than those reported here. Our 37 °C value is higher than the values attributed to Madsen [7]. Madsen’s two values for p*K*_sp_(OCP) are recalculations from his calculated solubility data that were based on an equilibrium model which did not include the ion pairs CaH_2_PO_4_^+^, CaHPO_4_^0^, and CaOH^−^. The Moreno et al. [8] value at 25°C is based on a single composition (the singular point for brushite and OCP). Thus, even though it has been recalculated to take into account calcium and phosphate ion pairs, relatively little reliance can be placed on this value [8]. We discuss the causes for the discrepancies between our p*K*_sp_(OCP) values and those of Shyu et al. [5] and of Heughebaert and Nancollas [6] in the following sections.

### Thermodynamic Systems and Ionic Model

Heughebaert and Nancollas [6] and Shyu et al. [5] used a five component system, Ca(OH)_2_–H_3_PO_4_–KNO_3_–H_2_O–KOH. The component KNO_3_ was included in the Heughebaert and Nancollas [6] and Shyu et al. [5] systems so that extrapolations could be made to zero ionic strength in evaluating p*K*_sp_(OCP). The component KOH was used to adjust the pH. We used mostly the ternary system, Ca(OH)_2_–H_3_PO_4_–H_2_O, except for the studies with initially supersaturated solutions. Our use of a ternary system was motivated by the desire to keep the system as simple as possible so as to avoid unanticipated ionic interactions. In the ternary system, the ionic strengths were generally so low that the ion activities could be calculated directly using the extended Debye-Hückel [10] or Davies equation [20]. However, both methods should yield satisfactory results provided they are based on a valid ionic model of the system. It is interesting that the p*K*_sp_(OCP) values are not significantly different when either the extended Debye-Hückel equation or Davies equation is used as shown in tables 1 and 8.

### Equilibration Conditions

Heughebaert and Nancollas [6] equilibrated their samples for 25 days at 25°C and 12 days at 45 °C, apparently without sampling at shorter time periods; Shyu et al. [5] sampled at termination of each experiment after periods of 4, 10, 30 or 40 days. As indicated in the Methods section, we used higher solid-to-solution ratios (10 mg/mL) as compared to Shyu et al. [5] and Heughebaert and Nancollas (3 mg/mL), and sampled frequently at much shorter time periods. In doing so, a constant value of p*K*_sp_(OCP) was obtained in a relatively short time. In the experiments with prolonged equilibration times, a subsequent increase in the value of the apparent p*K*_sp_(OCP) was observed which we attribute to hydrolysis of the OCP to a more basic and less soluble form of calcium phosphate. Table 2 clearly shows increases in the apparent p*K*_sp_(OCP) after the longer equilibration times.

Heughebaert and Nancollas [6] and Shyu et al. [5] approached equilibrium from both undersaturation and supersaturation. Most of our equilibrations were from undersaturation, but good agreement exists between our data from supersaturation and undersaturation.

The value of a p*K*_sp_(OCP) calculated from a set of experimental data is particularly sensitive to small errors in the measurement of pH. Glass electrodes were used by both groups of investigators. Heughebaert and Nancollas [6] and Shyu et al. [5] used a silver/silver chloride reference electrode which incorporated an intermediate liquid junction containing potassium chloride solution at the same ionic strength as the solution being studied and, therfore, avoiding the diffusion of the potassium chloride into solution. We used a standard calomel reference electrode and minimized the diffusion of the potassium chloride by avoiding prolonged contact between the electrode and the solution.

### Equilibrium Constants

The p*K*_sp_(OCP) is calculated from the pH, [Ca], and [PO_2_] data through the use of an ionic equilibrium model [15]. This model uses dissociation constants of H_3_PO_4_ and H_2_O and the association constants of the ion pairs CaHPO_4_, ${\text{CaH}}_{2}{\text{PO}}_{4}^{+}$ and Ca(OH)^+^ and, in this study, CaCO_3_ and CaHCO_3_. A comparison of the constants used by Heughebaert and Nancollas [6] with those used by us (shown in table 13) reveals substantial differences in the formation constants for the ion pairs at some of the temperatures. In table 1 are given the 25, 37 and 45°C values of p*K*_sp_(OCP) reported by Shyu et al. and Heughebaert and Nancollas [5,6]. Below each of these values is given in parentheses the value we calculated using their data for pH, [Ca], [PO_4_], and neutral ions with our model and equilibrium constants. The p*K*_sp_(OCP) values calculated by Heughebaert and Nancollas [6] are significantly higher than our values calculated from the same experimental data; these differences are probably due to the different equilibrium constants used, since both the extended Debye-Hückel equation and the Davies equation gave the same results. Clearly, there is a need to reassess the values of the ion pair formation constants since they contribute Significantly to the differences in the p*K*_sp_(OCP) values as shown in table 1.

The p*K*_sp_(OCP) values in our study are similar to those calculated from their data [5,6] with our model and equilibrium constants (table 1); our values are slightly lower than theirs at 23.5 to 37°C probably due to the hydrolysis of OCP in their study.

## Figures and Tables

**Table 1:** Solubility products of OCP, as p*K*_sp_(OCP), at various temperatures, this work and literature values. Values in parentheses were recalculated using our ionic equilibrium model

Temp. (°C)	This work	Nancollas et al. [5,6]	Madsen [7]	Moreno et a1. [8]
4	48.3±0.2			
4.8	48.3±0.2			
6.0	48.2			
18	48.3			
23.5	48.4±0.1			
25		49.6±0.2^a^		46.97^b^
		(49.0±0.2)^c^		
37	48.7±0.2	49.3±0.2^a^	48.46^a^	
		(49.1±0.3)^b^	(48.0)^b^	
45		49.8±0.3^a^		
		(48.6±0.4)^b^		

a Calculated by Shyu et al. [5] and Heughebaert and Nancollas [6].

b Recalculated by using our ionic models and constants.

c Recalculated by using our ionic constants; when ionic activity coefficients were calculated from the extension of the Debye-Hiickellimiting law, the p*K*=48.97±0.25; from the Davies equation p*K* =49.03±0.23.

**Table 2:** Various times of equilibration of two batches of OCP with 0.457 mmol/L H_3_PO_4_ solutions from undersaturation at 37 °C and under N_2_ atmosphere

Time of equilib.	pH	Composition of solution		$\frac{\text{Ca}}{P-P_{0}}$	p*IP*
		[Ca] mmol/L	[P] mmol/L		
OCP-A					

0	3.35	0.0	0.457		
15 min	6.35	0.614	0.994	1.14	48.86
31 min	6.32	0.616	0.993	1.15	48.98
62 min	6.30	0.630	1.032	1.10	48.98
2 h	6.36	0.644	1.052	1.09	48.68
4.5 h	6.35	0.670	1.092	1.06	48.68
23 h	6.16	0.806	1.367	0.89	48.88
10 d	5.43	2.91	5.37	0.59	48.78
2 months	4.75	7.29	13.91		49.69^a^
					av 48.83

OCP-B					

0	3.33	0.0	0.457		
15 min	6.38	0.685	0.959	1.36	48.61
33 min	6.37	0.703	0.982	1.34	48.58
65 min	6.40	0.694	0.972	1.35	48.65
2 h	6.41	0.701	0.996	1.30	48.38
4.5 h	6.43	0.685	0.969	1.34	48.36
23 h	6.33	0.767	1.070	1.25	48.50
10 d	6.10	1.022	2.912	0.42	48.32
2 months	4.96	6.57	13.52	0.50	48.84^a^
					av 48.49

a Were not included in solubility calculation.

**Table 3:** p*IP* of OCP from supersaturation at 37 °C

Time of equilib.	pH	Composition of solution		$\frac{\text{Ca}-{\text{Ca}}_{0}}{P-P_{0}}$	p*IP*
		[Ca] mmol/L	[P] mmol/L		
0	5.70	4.43	3.33		47.59
20 min	5.47	4.41	3.38	−0.40	48.68
1 h	5.46	4.37	3.36	−2.00	48.75
2 h	5.45	4.35	3.39	−1.33	48.80
4 h	5.43	4.42	3.43	−0.10	48.86
22 h	5.41	4.60	3.73	0.43	48.79
					av 48.78

0	5.70	4.80	3.28		47.50
42 min	5.35	5.53	4.00	1.01	48.71
2 d	5.30	5.61	3.98	1.16	48.94
3 d	5.27	6.02	4.44	1.05	48.87
5 d	5.22	6.67	4.95	1.12	48.83
12 d	5.07	8.42	6.77	1.04	48.86
					av 48.84

**Table 4:** p*K*_sp_ of OCP under 5.5 volume % of CO_2_/94.5 % N_2_ atmosphere from undersaturation and at 37 °C

Time of equilib.	pH	Composition of solution		$\frac{\text{Ca}}{P-P_{0}}$	p*K*_sp_
		[Ca] mmol/L	[P] mmol/L		
0	3.196	0	0.509		
1.5 h	5.996	1.27	1.53	1.24	48.80
2.0 h	5.937	1.31	1.57	1.23	49.00
28.0 h	5.928	1.30	1.69	1.10	48.97
2 d	5.949	1.30	1.73	1.06	48.84
5 d	5.914	1.50	1.99	1.01	48.61
7 d	5.930	1.49	2.04	0.97	48.52
9 d	5.812	1.63	2.15	0.99	48.87
12 d	5.764	1.80	2.65	0.84	48.70
16 d	5.675	1.95	2.99	0.79	48.86
					av 48.80

0	2.962	0	0.935		
1.5 h	5.808	1.42	2.07	1.25	49.14^a^
2.0 h	5.808	1.52	2.16	1.24	48.99
28.0 h	5.814	1.46	2.16	1.19	49.03
2 d	5.841	1.49	2.22	1.16	48.83
5 d	5.817	1.64	2.36	1.15	48.72
7 d	5.853	1.59	2.32	1.15	48.63
9 d	5.773	1.66	2.35	1.17	48.92
12 d	5.742	1.86	2.84	0.98	48.68
16 d	5.695	1.99	3.03	0.95	48.72
					av 48.82

a Was not included in solubility calculation.

**Table 5:** p*K*_sp_ of OCP under air atmosphere at 37 °C

Time of equilib.	pH	Composition of solution		$\frac{\text{Ca}}{P-P_{0}}$	p*K*_sp_
		[Ca] mmol/L	[P] mmol/L		
0		0	0.463		
0.5 h	6.35	0.606	1.073	0.99	48.80
1 h	6.33	0.621	1.083	1.00	48.84
2 h	6.32	0.650	1.083	1.05	48.81
28 h	6.10	1.056	1.756	0.82	48.44
					av 48.72

0			0.463		
2 h	6.33	0.630	1.139	0.93	48.74
2 d	5.83	1.384	2.500	0.68	48.86
3 d	5.68	1.74	3.424	0.59	48.86
					av 48.82

**Table 6:** Solubility of OCP at 4 °C

Time of equilib. (days)	pH	Composition of solution		$\frac{\text{Ca}}{P-P_{0}}$	p*K*_sp_
		[Ca] mmollL	[P] mmol/L		
0		0	0.236		
1	7.11	0.459	0.613	1.22	48.37
4	7.11	0.478	0.618	1.25	48.46
5	7.10	0.471	0.64	1.17	48.48

0		0	0.543		
5	6.77	0.781	1.22	1.12	48.18

0		0	0.825		
11	6.63	0.980	1.657	1.18	48.03

0		0	0.894		
1	6.52	1.05	1.401	2.07	48.57
2	6.54	1.05	1.667	1.36	48.29
4	6.55	0.989	1.664	1.28	48.34
					av 48.26±0.19

**Table 7:** Solubility of OCP at 4.8 °C

Initial conditions			Time of equilib. (days)	pH	Composition of solution		$\frac{\text{Ca}-{\text{Ca}}_{0}}{P-P_{0}}$	p*K*_sp_
pH	H_3_PO_4_ mmol/L	Ca(OH)_2_ mmol/L			[Ca] mmol/L	[P] mmol/L		
	0.248	0	11	6.93	0.577	0.791	1.06	48.47
	0.543	0	1	6.77	0.765	1.23	1.11	48.16
	0.849	0	17	6.38	1.17	2.05	0.97	48.52
	1.7	0	17	6.14	1.75	3.21	1.16	48.46
6.08	1.48	0.79	12	6.44	1.06	2.50	0.26	48.20
5.62	3.0	1.61	12	6.15	1.95	3.31	1.10	48.21
								av 48.34±0.16

**Table 8:** Solubility of OCP at 6.0 °C

Time of equilib.	pH	Composition of solution			
		[Ca] mmol/L	[P] mmol/L	p*K*_sp_	
				Ext. DH	Davies
0	5.92	4	3		
5 min	5.95				
2 h	5.95	4.19	3.15	48.15	48.19
1 d	5.97	4.01	3.14	48.13	
4 d	5.95	3.93	3.11	48.26	
7 d	5.97	3.836	3.05	48.30	48.34
				av 48.21±0.08	

**Table 9:** Solubility of OCP at 18 °C

Time of equilib. (days)	pH	Composition of solution		p*K*_sp_
		[Ca] mmol/L	[P] mmol/L	
9	6.76	0.586	0.897	48.24
22	6.47	0.840	1.486	48.27

**Table 10:** Solubility of OCP at 23.5 °C

Initial conditions			Time of equilib. (days)	pH	Composition of solution		$\frac{\text{Ca}-{\text{Ca}}_{0}}{P-P_{0}}$	p*K*_sp_
pH	H_3_PO_4_ mmol/L	Ca(OH)_2_ mmol/L			[Ca] mmol/L	[P] mmol/L		
	0.849		17	6.09	1.62	2.04	1.36	48.31
	1.7		17	5.94	1.74	3.20	1.16	48.37
	3.39		12	5.57	2.94	5.87	1.19	48.67
6.08	1.48	0.79	12	6.26	0.99	1.92	0.45	48.40
5.62	3.0	1.16	12	5.92	1.69	3.60	0.13	48.40
								av 48.43±0.14

**Table 11:** p*K*_sp_ of OCP under N_2_ atmosphere at 37 °C

Time of equilib.	pH	Composition of solution		$\frac{\text{Ca}}{P-P_{0}}$	p*K*_sp_
		[Ca] mmol/L	[P] mmol/L		
0		0	0.242		
2 h	6.75	0.331	0.524	1.174	48.93
2 d	6.45	0.549	0.941	0.785	48.66
6 d	6.10	0.876	1.64	0.626	48.82
					av 48.80

0		0	0.463		
2 h	6.33	0.633	1.07	1.048	48.80
6 h	6.28	0.644	1.15	0.967	48.86
11 h	6.14	0.792	1.41	0.840	48.97
1 d	6.03	0.989	1.77	0.758	48.86
2 d	5.64	1.93	3.71	0.594	48.80
					av 48.86

0	2.96	0	0.935		
2 h	5.81	1.52	2.16	1.241	48.99
1 d	5.81	1.46	2.16	1.192	49.03
2 d	5.84	1.49	2.22	1.160	48.83
5 d	5.82	1.64	2.36	1.151	48.72
7 d	5.85	1.59	2.32	1.148	48.63
					av 48.84

0	6.08	0.79	1.48		
12 d	6.26	0.99	1.920	0.45	48.45

0	5.62	1.61	3.00		
12 d	5.70	1.95	4.28	0.27	48.44

0		0	3.39		
12 d	5.52	2.76	6.02	1.05	48.40

0		0	3.50		
2 h	5.37	2.86	5.67	1.32	49.02^a^
1 d	5.42	2.95	5.72	1.33	48.72
2 d	5.33	3.33	6.47	1.12	48.83
3 d	5.31	3.41	6.68	1.07	48.86
7 d	5.24	4.32	8.47	0.87	48.59
					av 48.75

a Was not included in solubility calculation.

**Table 12:** Calculated ΔCa, ΔP, and ΔCa/AP values from the 25 °C data of Heughebaert and Nancollas [6]. The equilibrations 44G through 52G are equilibrations approached from supersaturation; those labeled 44D to 52D are from undersaturation

*#*	ΔCa	ΔP	ΔCa/ΔP
52G	0.014	0.005	2.8
50G	0.20	0.016	1.25
48G	0.37	0.22	1.68
47G	0.26	0.20	1.30
46G	0.27	0.15	1.80
44G	0.18	0.11	1.64
52D	0.092	0.096	0.96
50D	0.157	0.185	0.85
48D	0.151	0.187	0.81
47D	0.197	0.206	0.96
46D	0.215	0.227	0.95
44D	0.266	0.236	1.13

**Table 13:** Equilibrium constants [5,6,15]

	25 °C		37 °C		45°C	
	This study	Heughebaert et al. [6]	This study	Shyu et al. [5]	This study	Heughebaert et al. [6]
H_3_PO_4_⇌H^+^ +H_2_PO_4_^−^	7.11 × 10^−3^	7.11 × 10^−3^	6.22 × 10^−3^	6.22 × 10^−3^	5.63 × 10^−3^	5.63 × 10^−3^
H_2_PO_4_⇌H^+^ +HPO_4_^2−^	6.31 × 10^−3^	6.30 × 10^−8^	6.58 × 10^−8^	6.58 × 10^−8^	6.60 × 10^−8^	6.61 × 15^−8^
HPO_4_^2−^⇌H^+^ +PO_4_^3−^	4.52 × 10^−13^	4.73 × 10^−13^	6.84 × 10^−13^	6.61 × 10^−13^	9.02 × 10^−13^	6.61 × 10^−13^
Ca^2+^ + H_2_PO_4_^−^⇌CaH_2_PO_4_^+^	8.48	25.6	7.01	31.9	5.57	36.5
Ca^2+^ +HPO_4_^2−^⇌CaHPO_4_	264	548	355	681	503	787
Ca^2+^ + PO_4_^3−^⇌CaPO_4_^−^	2.9 × 10^6^	2.9 × 10^6^	2.9 × 10^6^	3.46 × 10^6^	2.9 × 10^6^	3.86 × 10^6^
Ca^2+^ +OH⇌CaOH^+^	20.0	13.8	20.0	21.3	20.0	28.4
H^+^ +OH^−^⇌H_2_O	1.013 × 10^−14^	1.004 × 10^−14^	2.41 × 10^−14^	2.42 × 10^−14^	4.04 × 10^−14^	4.09 × 10^−14^

## Footnotes

1 Certain commercial materials and equipment are identified in this paper to specify the experimental procedure. In no instance does such identification imply recommendation or endorsement by the National Bureau of Standards or the ADA Health Foundation or that the material or equipment identified is necessarily the best available for the purpose.

## Appendix

The ionic activity coefficients were calculated from the extension of the Debye-Hückel limiting law [19] or Davies equation [20]. The expanded Debye-Hückel equation is

$$\log\;f_{i}=-Az^{2}\surd I/(1+Ba_{i}\surd I)$$

and the Davies equation is

$$\log\;f_{i}=-Az^{2}[\surd I/(1+\surd I)-0.3I],$$

where *f*_i_ and *I* are the activity coefficients of species i and the ionic strength, respectively. *A* and *B* are functions of the temperature and the dielectric constant of the solvent (Appendix table 4, ref. [19]). For the parameters *a*_1_ we used for Ca^++^: 6×10^−8^ cm; for H_2_PO_4_^−^: 4.5 × 10^−8^ cm; for HPO_4_^=^ and PO_4_^=^: 4×10^−8^ cm; for H^+^: 9× 10^−8^ cm; and for OH^−^: 3.5× 10^−8^ cm [21]. The use of different values of *a*_i_ thermodynamically violates the Gibbs-Duhem equation [22]. The equilibrium constants for ion pairs, phosphoric acid and water are shown in table A1 [15,16,22]: log_10_*k* =*A*_1_/*T*^2^+*A*_2_/*T* +*A*_3_+*A*_4_*T* + *A*_5_log_10_*T*, where *T*=kelvins. The temperature dependence of the *k*_3_ value for H_3_PO_4_ is given by: −log_10_*k* = 12.45 −0.015(°C–18.0), where °C is the temperature.

**Table A1:** Parameters for equilibrium constants

Ion pair	*A*_1_	*A*_2_	*A*_3_	*A*_4_	*A*_5_
H_3_PO_4_ *k*_1_	0.	−799.31	4.5535	−0.01349	0.
H_3_PO_4_ *k*_2_	0.	−1989.32	5.4374	−0.02001	0.
*k*_w_	0.	−4470.99	6.0875	−0.01706	0.
H_2_CO_3_ *k*_1_	−1684915.	21834.37	−356.3094	−0.06092	126.8339
H_2_CO_3_ *k*_2_	−563714.	5151.79	−107.8871	−0.03253	38.9256
H_2_CO_3_ *k*_H_ (gas partition)	669365.	−6919.53	108.3865	0.01985	−40.4515
CaH_2_PO_4_	0.	−8232.51	57.1388	−0.09592	0.
CaHPO_4_	0.	12213.79	−81.1027	0.14275	0.
CaPO_4_	0.	0.	6.4624	0.	0.
CaOH	0.	0.	1.3010	0.	0.
CaHCO_3_	0.	−34765.05	1209.1200	0.31294	478.782
CaCO_3_	0.	35512.75	−1228.7320	−0.29944	−485.818
